# Intravascular Large B-cell Lymphoma Associated With Hemophagocytosis as a Cause of Fever of Unknown Origin: A Review of Literature

**DOI:** 10.7759/cureus.73385

**Published:** 2024-11-10

**Authors:** Shula Staessens, Nathan De Beule, Els Van Nedervelde, Sabine Allard

**Affiliations:** 1 Internal Medicine, Universitair Ziekenhuis Brussel, Brussels, BEL; 2 Clinical Hematology, Universitair Ziekenhuis Brussel, Brussels, BEL; 3 Internal Medicine and Infectiology, Universitair Ziekenhuis Brussel/Vrije Universiteit Brussel, Brussels, BEL; 4 Neuro-Aging and Viro-immunotherapy Research Group (NAVI), Vrije Universiteit Brussel, Brussels, BEL; 5 Internal Medicine and Infectiology, Universitair Ziekenhuis Brussel, Brussels, BEL

**Keywords:** deep skin biopsy, fever of unknown origin (fuo), intravascular large b-cell lymphoma, lymphohistiocytic hemophagocytosis, secondary hlh

## Abstract

A 53-year-old woman was hospitalized due to a fever of unknown origin for three weeks. Given the presence of fever and fatigue, the laboratory findings, and a bone marrow biopsy confirming hemophagocytic lymphohistiocytosis (HLH), a hematological malignancy was suspected. Peripheral lymphocytic typing, bone marrow biopsy, and imaging could not identify an underlying cause of HLH. Therefore, an abdominal wall deep skin biopsy was performed, showing an intravascular large B-cell lymphoma (IVLBCL). Intravascular large B-cell lymphoma should be considered in all patients with fever of unknown origin, especially in the context of HLH. Diagnosis of IVLBCL requires a deep skin biopsy or a biopsy of an affected organ.

## Introduction

Intravascular large B-cell lymphoma (IVLBCL) is a rare disease that is difficult to diagnose and has a poor clinical outcome. It can present with a wide variety of symptoms due to the possible involvement of multiple organs [1]. Based on clinical and histological manifestations, IVLBCL can be divided into two main variants that present with systemic symptoms: the classical and the hemophagocytic syndrome-associated variant. There is also a third clinically relevant variant known as the cutaneous variant. The classical variant generally presents with neurological symptoms and skin lesions, whereas the hemophagocytic syndrome-associated variant is, as the name suggests, accompanied by hemophagocytic syndrome or hemophagocytic lymphohistiocytosis (HLH). The cutaneous variant has the histological features of the classical variant, but symptoms are limited to the skin [2,3]. We present a rare case of IVLBCL associated with HLH.

## Case presentation

A 53-year-old Caucasian woman with no medical history was hospitalized due to a fever of up to 40 °C and fatigue for three weeks. She reported a similar episode of fatigue but without fever four months prior, which lasted a few weeks and resolved spontaneously.

Clinically, vitals were hypotension (85/55 mmHg), fever up to 39 °C, generalized pitting edema, and exercise dyspnea (NYHA II-III). There was no skin rash. On admission, laboratory analysis (Table 1) showed high inflammatory markers, high lactate dehydrogenase (LDH), hypertriglyceridemia, normocytic anemia without signs of hemolysis (normal bilirubin level, normal haptoglobin, and absence of schistocytes), hypoalbuminemia without proteinuria and a normal white blood cell count with monocytosis. Initially, the patient had a normal platelet count, but she developed thrombocytopenia during hospitalization. Ferritin levels were elevated, starting at 1365 µg/L but reaching a maximum of 6974 mg/L. Fibrinogen levels were normal on admission but dropped below 250 mg/dL during hospitalization. The kidney function and electrolytes were within normal limits. Hormonal analysis turned out to be normal as well.

**Table 1:** Biochemistry analysis on admission

Parameter	Value	Reference
Creatinine	0.78 mg/dL	0.52–1.04 mg/dL
Estimated glomerular filtration rate (eGFR)	87 ml/min/1.73 m²	>60 ml/min/1.73 m²
Albumin	21 g/L	35–50 g/L
C-reactive-protein (CRP)	120.3 mg/L	<5 mg/L
Lactate dehydrogenase	2087 U/L	313–618 U/L
Hemoglobin	9.2 g/dL	11.8–14.5 g/dL
Thrombocytes	187 x 10³/mm³	158–450 x 10³/mm³
Leucocytes (absolute count)	6.2 x 10³/mm³	3.6–9.6 x 10³/mm³
Monocytes (absolute count)	1.551 x 10³/mm³	0.1–1.0 x 10³/mm³
Ferritin	1365 µg/L	13–150 µg/L
Triglycerides	507 mg/dL	<150 mg/dL

Infectious causes, such as Mycobacterium tuberculosis, SARS-CoV-2, toxoplasmosis, Cytomegalovirus, Epstein-Barr virus, brucellosis, Bartonella, syphilis, borreliosis, Parvovirus B19, leishmaniasis, histoplasmosis and rickettsiosis, and rheumatological conditions were ruled out. Serum protein electrophoresis was normal, and peripheral lymphocytic typing showed no signs of monoclonality.

**Figure 1:**
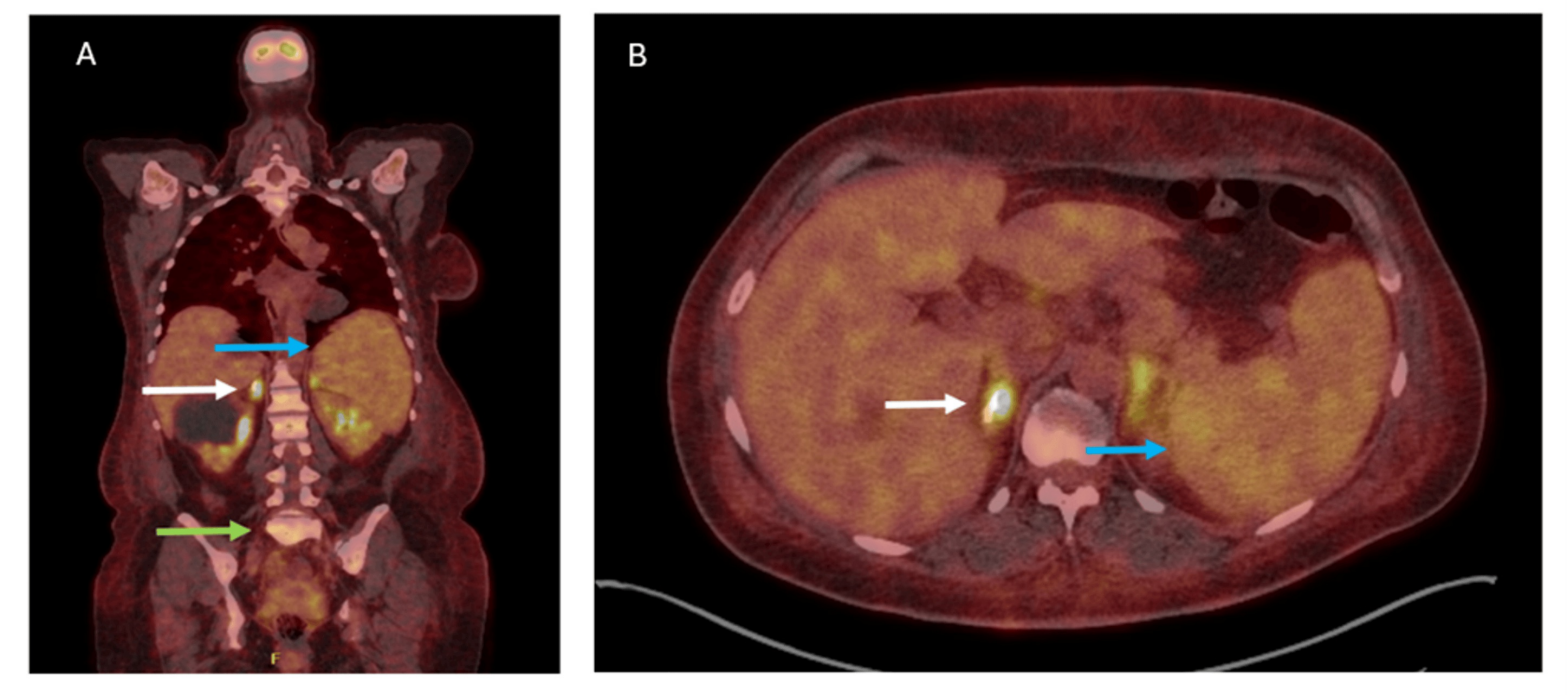
PET-CT findings in our patient Increased 18F-fluorodeoxyglucose uptake in the adrenal glands (white arrows), bone marrow (green arrow), and spleen (blue arrows). (A) Coronal fused view and (B) axial fused view.

A computed tomography (CT) scan of the abdomen showed hepatosplenomegaly as well as pericardial and pleural effusion. The adrenal glands, bone marrow, and spleen showed increased 18F-fluorodeoxyglucose (FDG) uptake on the positron emission tomography (PET) scan (Figure 1). There were no significant lymphadenopathies. A CT scan of the brain, as well as additional magnetic resonance imaging (MRI), showed two lesions, frontal and occipital, on the right side of the brain, suggestive of meningiomas.

With the presence of hypertriglyceridemia and hypofibrinogenemia, bicytopenia, hyperferritinemia, splenomegaly, and fever, five out of eight criteria for HLH were met according to the HLH-2004 protocol. With the fulfillment of these parameters, the diagnosis of HLH had already been made [4]. The H-score at that moment was 258 points with a probability of hemophagocytic syndrome of more than 99%. In addition, a bone marrow biopsy was performed [5].

Immunophenotyping showed an abnormally high percentage of CD16+ monocytes (>50% of monocytes), which were positive for HLA-DR, CD15, CD33, CD45, and CD11c and negative for CD11b and CD14. This can be seen in hemophagocytosis and can thus be compatible with HLH [6]. The anatomo-pathological report also showed signs of hemophagocytosis. Given the age of the patient, a secondary cause of HLH was suspected. The patient's condition remained stable, and we delayed initiating HLH treatment until we conducted additional tests to identify any underlying conditions. A second bone marrow biopsy was performed 14 days after the first one. This second biopsy showed that more than 80% of monocytes were CD16+. Both biopsies showed a normal percentage of T- and B-lymphocytes. The anatomo-pathological report of both biopsies stated a hypercellular bone marrow with macrophage activity and T-cell stimulation compatible with the hemophagocytic syndrome without signs of monoclonality.

By the time the second biopsy was performed, analysis of natural killer cell activity and soluble interleukin-2 receptor (sIL-2r or sCD25) had been done. Natural killer cell activity was normal. Soluble CD25 was elevated (41,485 pg/mL; normal value <2,000 pg/mL). The diagnosis of hemophagocytic syndrome had already been retained, but now seven out of eight criteria had been fulfilled [4]. However, as of yet, there is still no identification of an underlying etiology.

**Figure 2:**
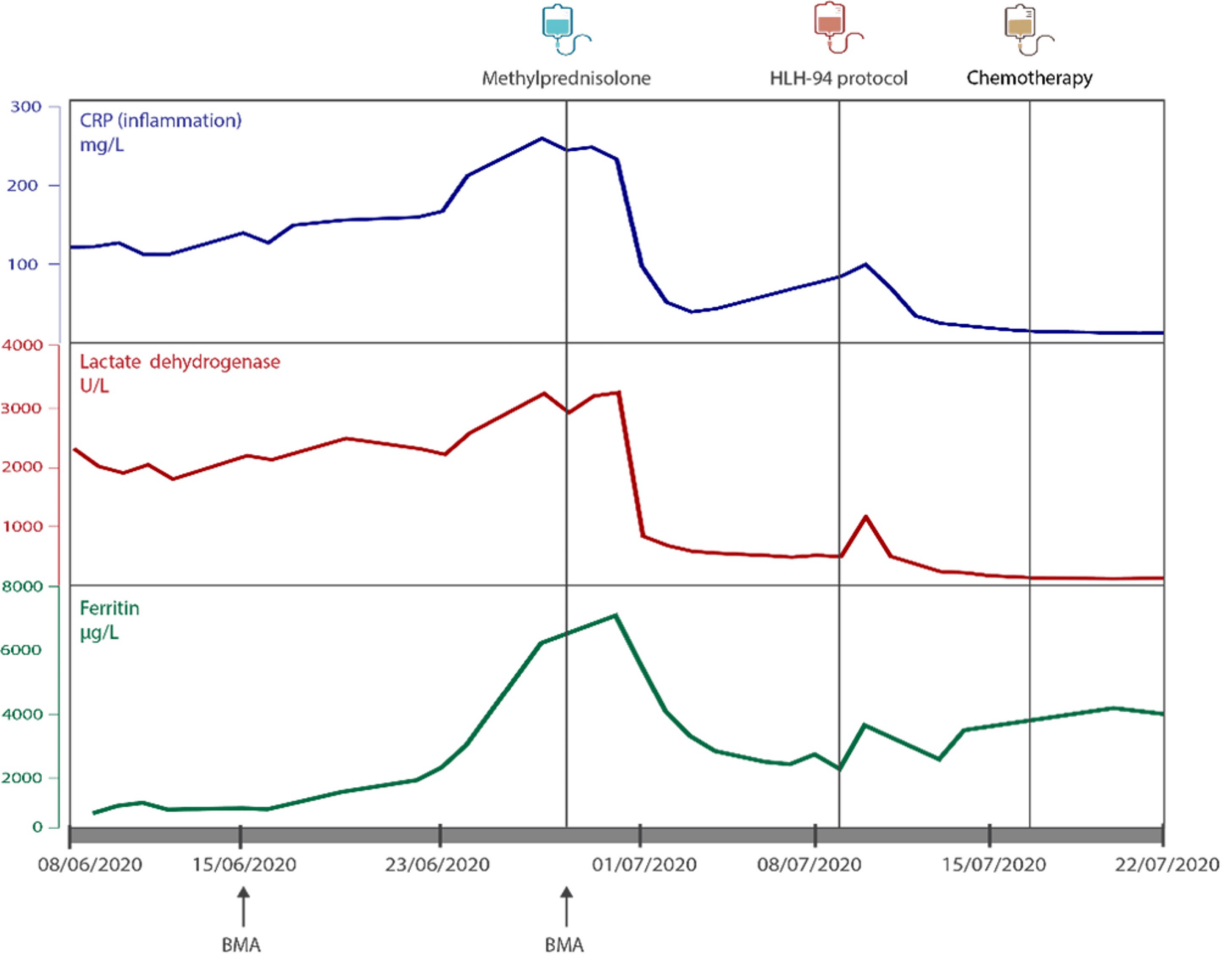
Evolution of CRP, LDH, and ferritin BMA: bone marrow aspiration and biopsy. Chemotherapy: R-CHOP.

Both clinically and biochemically, the patient’s condition was starting to deteriorate. She was started on treatment with corticosteroids, intravenous methylprednisolone 1 g once daily for three days, followed by methylprednisolone 64 mg once daily. Initially, some improvement was seen with this therapy. There was no fever for four days, and LDH, ferritin, and CRP levels were decreasing (Figure 2). However, fever relapsed, and in accordance with the HLH-94 protocol, treatment with dexamethasone 10 mg/m² and etoposide 150 mg/m² was started [7]. Cyclosporin A was not given due to the risk of posterior reversible encephalopathy syndrome and due to an epileptic seizure during this hospitalization. Biochemically, some improvement was seen with this treatment, but values did not return to normal (Figure 2), probably given the disease-causing abnormalities had not been found and treated.

As the more common causes of fever of unknown origin/HLH had been ruled out, we decided to perform a deep skin biopsy of the abdominal wall in seemingly normal skin. This biopsy showed a large population of abnormal cells (CD20 and Pax5 positive) as well as CD163 histiocytes in the small vessels. These abnormal B cells expressed Bcl6/mum1 and were negative for Bcl2 and cMyc, which was compatible with the diagnosis of an IVLBCL.

Consequently, the patient was treated with R-CHOP chemotherapy, which comprised rituximab (cycle 1 375 mg/m², cycle 2-1400 mg), cyclophosphamide 750 mg/m², doxorubicin hydrochloride 50 mg/m², vincristine 1.4 mg/m², and prednisone 60 mg/m². Initially, according to the HLH-94 protocol, intrathecal methotrexate (MTX) 12 mg was given [4]. However, following the confirmation of IVLBCL diagnosis, invasion of the central nervous system (CNS) had not been ruled out yet. Taking into account the two brain lesions, which were compatible with meningiomas on imaging but not yet histologically confirmed, along with monocytosis detected in the cerebrospinal fluid, high-dose IV MTX at 3000 mg/m² was started. The evolution of the CRP, LDH, and ferritin levels during these treatments can be seen in Figure 2.

Despite completing four of the six cycles of chemotherapy, residual hemophagocytosis was still present on a bone marrow biopsy, and brain lesions did not regress in volume. Therefore, a brain biopsy was performed, confirming the presence of two meningiomas and, thus, no evidence of CNS invasion by the IVLBCL. Because of residual hemophagocytosis on bone marrow biopsy and thus suspicion of refractory disease, the treatment was intensified with two cycles of R‑DHAP: rituximab 1400 mg, dexamethasone 40 mg, cytarabine 2000 mg/m², cisplatin 100 mg/m², followed by an autologous stem cell transplant.

Following stem cell transplantation, the patient had a slow hematological recovery. Due to the development of secondary humoral immune deficiency after stem cell transplantation, monthly administration of intravenous immunoglobulins (IVIg) was started. As hemophagocytosis was still present on bone marrow biopsy three months after stem cell transplantation, dexamethasone 10 mg every two days was given. A skin biopsy performed at that point showed no signs of lymphoma. After reducing the dose, cytopenias returned, and thus, 100 mg of etoposide once daily was added five months after stem cell transplantation. Six months after the autologous stem cell transplant, the PET-CT scan, skin biopsies, and bone marrow biopsy revealed no evidence of lymphoma or hemophagocytosis.

Persistent steroid-dependent cytopenias were then treated with granulocyte colony-stimulating factor, erythropoietin stimulating agents, and thrombopoietin receptor agonists, as well as Deferasirox to treat the iron overload following multiple transfusions with a favorable outcome. To this day, three years after autologous stem cell transplant, the patient still receives monthly administration of IVIg. As of yet, there are no signs of relapsed lymphoma. Clinically, she made a full recovery.

## Discussion

Epidemiology and clinical presentation

IVLBCL is a rare clinical entity characterized by malignant B-lymphocyte growth in the lumina of small-sized blood vessels, mostly capillaries and post-capillary venules [2]. The incidence is estimated to be around one in a million, though the true incidence is unknown [1]. The median age at diagnosis is 70 years old. There is no sex prevalence [3,8]. The World Health Organization (WHO) classifies IVLBCL as a subtype of large B-cell lymphoma’s [9]. Within the IVLBCL, we identify two main variants, the classical and the hemophagocytic, historically known as the “Western” and the “Asian” variants. However, variants are no longer referred to as such because of the overlap between the two variants and the geographical distribution [2,10,11]. The Western variant is characterized by cutaneous and neurological involvement, whereas the Asian variant is known for its association with hemophagocytic syndrome. Current WHO classification is based on histology and clinical presentation, i.e., classical or hemophagocytic syndrome-associated variant. A third variant, the cutaneous variant, is characterized by skin lesions without systemic involvement [8].

The two main variants share some features, such as the presence of B-symptoms (present in 55-76% of cases), anemia (present in 63% of cases), and an increase of serum lactate dehydrogenase (present in 86% of cases). Usually, there is no lymphadenopathy; it is seen in only 11% of cases. They differ in the main sites of disease. In the hemophagocytic syndrome-associated variant, the liver, spleen, and bone marrow are often involved. This is in contrast to the classical variant, where the skin and the central nervous system are the most affected locations [3,11-13]. Frequent manifestations that are seen in the hemophagocytic syndrome-associated variant and not in the classical variant are thrombocytopenia and hypoalbuminemia [3,11,12]. The patient in our case presented with the hemophagocytic syndrome-associated IVLBCL variant.

The Classical IVLBCL Variant

Patients with the classical variant often present with cutaneous and/or neurological symptoms. Cutaneous symptoms are present at diagnosis in 40% of cases. A wide variety of skin lesions can be seen in patients with IVLBCL, such as cellulitis, “peau d’orange,” erythematous plaques, nodules, and others. Neurological symptoms are present in 35% of patients. These symptoms are heterogeneous and include neuropathies, aphasia, dysarthria, and seizures, amongst various others. Patients can also present with organ-specific symptoms [3,13,14].

The Hemophagocytic Syndrome-Associated IVLBCL Variant

The patient described in this case presented with the hemophagocytic syndrome-associated variant of IVLBCL. Histologically, this differs from the classical variant by the presence of histiocytes with phagocytic activity in the peripheral blood or bone marrow aspirate [3,8,10].

The diagnostic criteria for hemophagocytic syndrome, also known as HLH, were determined by the Histiocyte Society in 1991. These include fever, splenomegaly, at least two out of three cytopenias, hypertriglyceridemia, and HLH, which were confirmed on the bone marrow biopsy. Three more criteria were added by Henter et al. in 2004 (Table 2). These are hyperferritinemia, low or absent NK cell activity, and high levels of sCD25. In order to confirm the diagnosis of HLH, five out of eight of these criteria are to be met [4].

**Table 2:** Diagnostic criteria according to HLH-2004 protocol HLH-2004 protocol [4].

Diagnostic criteria	Definition
Fever	More than 38.5 °C
Splenomegaly	
Cytopenias (≥2 out of 3 lineages)	Hemoglobin <9 g/dL, platelets <100 × 10³/mm³, neutrophils <1.0 × 10³/mm³
Hypertriglyceridemia and/or hypofibrinogenemia	Fasting triglycerides >265mg/dL, fibrinogen <150 mg/dL
Hemophagocytosis in bone marrow or spleen or lymph nodes	No evidence of malignancy
Low or absent NK-cell activity	According to laboratory reference
Hyperferritinemia	>500 µg/L
High sCD25	>2400 U/mL

Two forms of HLH are distinguished. The primary form results from a genetic disorder called familial hemophagocytic lymphohistiocytosis. In the secondary form, activation of the immune system occurs in reaction to other conditions, such as malignancies, infections, rheumatoid disorders, and genetic conditions [4]. In the event of the diagnosis of HLH, it is, therefore, essential to rule out an underlying condition.

In our patient, the diagnosis of HLH was retained as seven out of eight criteria were present. Suspecting the secondary form of HLH workup for malignancies, infectious diseases, and autoimmune diseases was performed. After the diagnosis of IVLBCL was made, the diagnosis of the secondary form of HLH, malignancy-related, was confirmed.

The Cutaneous IVLBCL Variant

The cutaneous variant is more common in Western countries (25% of all IVLBCL cases) compared to Asian countries (3% of all IVLBCL cases) [2,3]. Women also experience it more frequently and have a better prognosis due to the absence of systemic involvement. The median age at diagnosis is also lower, 59 years of age, compared to the classical and hemophagocytic variants. This variant presents with skin lesions, solitary or multiple lesions, with varying morphology and distribution patterns [3,8,13].

Diagnosis

IVLBCL can be diagnosed by a random deep skin biopsy or a biopsy of an affected organ. A Japanese study showed that the sensitivity and specificity of a random skin biopsy are around 77.8% and 98.7%, respectively [15]. An American and a Canadian study showed lower sensitivities and specificities [16,17]. However, it is critical to consider which method is used for biopsy, as punch biopsies might not comprise subcutaneous fat tissue. Most patients with IVLBCL lesions in the skin also have an invasion of the subcutaneous fat, but not the other way around. The presence of IVLBCL lesions in the subcutaneous fat without skin involvement is the case in up to 50% of the patients [15]. It is recommended to perform multiple deep skin biopsies of both skin lesions as well as seemingly unaffected skin, given that IVLBCL lesions can be found between normal skin and fat tissue [14].

Treatment

In the case of lymphoma-related HLH, the hemophagocytic syndrome is treated by treating the underlying disease. Currently, there are no evidence-based guidelines for the stage and treatment of IVLBCL. Therefore, treatment is based on guidelines for diffuse large B-cell lymphoma. Survival rates of IVLBCL have improved significantly with the introduction of immunotherapy such as rituximab. Given the aggressive nature of this disease, therapy consists of systemic treatment with immunotherapy in combination with chemotherapy. The most commonly used regimen is R-CHOP [3,8,11,18]. In cases of CNS involvement, methotrexate (MTX), cytarabine, and prednisolone can be added. MTX prophylaxis can also be added in patients without CNS involvement [19]. There is also a place for an autologous stem cell transplant in the treatment of an IVLBCL. However, given the rarity of the disease, this has not been extensively studied. The number of case reports or case series is limited, and there are no large randomized studies. An autologous stem cell transplant is an option, but given the median age of onset at 70 years old and the generally poor condition of the patients, this is not always possible. Some case reports did show a clinical improvement after high-dose chemotherapy followed by an autologous stem cell transplant compared to the use of R-CHOP, but further investigation is necessary [8,20].

Prognosis

Following treatment, the two-year overall survival rate of IVLBCL was reported to be 66%, with a progression-free survival of 56% [18]. This means early diagnosis is key. The difficulty, however, lies in diagnosing IVLBCL, given the rarity of involvement of peripheral blood (approximately in 5-9% of the cases) and bone marrow (up to one-third of the cases), the non-specific symptoms, and the rarity of the disease. Symptoms can mimic various conditions, depending on the organ affected [12,13,18].

Due to its difficult diagnosis and high mortality rate, the condition is more often identified post-mortem. It is reported to be diagnosed on autopsy in up to half of the patients [11,13]. Recent reviews, however, reported ante-mortem diagnoses up to 80%, possibly due to better awareness and recognition of the disorder [8,17]. Poorer outcomes were seen in patients with the hemophagocytic syndrome-associated variant [3].

## Conclusions

IVLBCL is a heterogeneous disease that can present with a variety of symptoms. It is difficult to diagnose and has a poor prognosis. Due to the rarity of this disease, the literature is limited to case reports and reviews of case studies. A better understanding of IVLBCL will lead to faster recognition and diagnosis, which will, in turn, result in quicker initiation of treatment, possibly improving the prognosis. Further research is needed to comprehend this disorder better. IVLBCL should be considered in all patients who present with a fever of unknown origin. With this report, we want to emphasize that diagnosis is often based on deep skin biopsy due to lack of peripheral blood or bone marrow involvement.
